# Chronic unpredictable stress exacerbates surgery-induced sickness behavior and neuroinflammatory responses via glucocorticoids secretion in adult rats

**DOI:** 10.1371/journal.pone.0183077

**Published:** 2017-08-14

**Authors:** Na Wang, Hong Ma, Zhe Li, Yalei Gao, Xuezhao Cao, Yanhua Jiang, Yongjian Zhou, Sidan Liu

**Affiliations:** Department of Anesthesiology, The First Hospital of China Medical University, Shenyang, Liaoning, China; Universidade de Sao Paulo, BRAZIL

## Abstract

Accumulated evidence indicates that stress sensitizes neuroinflammatory responses to a subsequent peripheral immune challenge. The present study investigated whether chronic unpredictable stress (CUS) aggravated surgery-induced sickness behavior and neuroinflammatory processes via glucocorticoids secretion in the adult brain.

**Methods:**

Sprague-Dawley adult male rats (12–14 weeks old) were exposed to 14-day CUS and then subjected to partial hepatectomy 24 h after the last stress session. The rats were pretreated with an antagonist of the glucocorticoids (GCs) receptor RU486 (30 mg/kg, i.p.) 1 h prior to stress exposure. The behavioral changes were evaluated with open field test and elevated plus-maze test. The hippocampal cytokines interleukin (IL)-1β and IL-6 were measured on postoperative days 1, 3 and 7. Ionized calcium binding adaptor protein (Iba)-1, microglial M2 phenotype marker Arg1, brain derived neurotrophic factor (BDNF) and CD200 were also examined at each time point.

**Results:**

CUS exacerbated surgery-induced sickness behavior. Exposure to CUS alone failed to alter the levels of pro-inflammatory cytokines in the brain. However, CUS exaggerated surgery-induced pro-inflammatory cytokines expression (e.g. IL-1β and IL-6) and upregulated the levels of Iba-1 on postoperative days 1 and 3. An additional significant decreased BDNF, CD200 and a lower level of Arg1 were also observed in the stressed rats following surgical procedure. Pretreatment with RU486 blunted the potentiating effects of CUS on surgery-induced sickness behavior and neuroinflammatory responses.

**Conclusion:**

Chronic unpredictable stress enhanced surgery-induced sickness behavior and neuroinflammatory responses. Stress-induced GCs played a pivotal role in enhancing surgery-induced neuroinflammatory processes by modulation of microglia functions.

## Introduction

Postoperative cognitive dysfunction (POCD), characterized by the progressive deterioration of intellectual/cognitive function, is a major complication following various surgical procedures [1]. Mounting evidence indicates that POCD is associated with surgery-induced neuroinflammatory processes, which may influence neuronal functioning either directly or through modulation of intraneuronal pathways, such as brain derived neurotrophic factor (BDNF) mediated pathway [2, 3]. High levels of neuroinflammatory cytokines, such as interleukin (IL)-1β, IL-6, and tumor necrosis factor (TNF)-α play a pivotal role in surgery-induced cognitive deficits [1, 4].

Acute and chronic stress sensitized or primed neuroinflammatory responses to both peripheral and central immunologic challenges [5, 6]. For example, chronic unpredictable stress (CUS) potentiated LPS-induced pro-inflammatory mediators (e.g., IL-1β, inducible nitric oxide synthase, and TNF-α) in frontal cortex and hippocampus of rats [7]. Interestingly, treatment with exogenous glucocorticoids (GCs) is sufficient to replicate the phenomenon of stress-induced priming of neuroinflammatory responses to peripheral immune challenges [8]. Furthermore, pretreatment with glucocorticoid receptors (GR) antagonist RU486 blunted the potentiating effects of stress on nuclear factor kappa B (NF-κB) expression [8, 9].

Surgical trauma caused sickness behavior and triggered neuroinflammatory responses in the brain of rats [1, 4, 10]. Psychological stress is common prior to the major surgery. It was reported to affect 60–80% of surgical patients [11]. The main objective of this study was to investigate whether CUS aggravated surgery-induced sickness behavior and neuroinflammatory responses in the adult rats. We also explored whether stress and the consequent increase of circulating GCs modulated the immunophenotype of microglia, thereby sensitizing neuroinflammatory responses to the subsequent surgical challenge.

## Methods

### Animals

Sprague-Dawley adult male rats (12–14 weeks old) were randomly divided into a total of six groups: control group (n = 30), CUS group (n = 36), RU486 group (n = 30), surgery group (n = 30), CUS+surgery group (n = 30), and RU486+CUS+surgery group (n = 30). All animals were housed in groups of four per cage except the day of surgery and had free access to food and water. Colony conditions were maintained at 25°C on a 12-h light/dark cycle (lights on at 07:00 A.M.). All rats were adapted to their environment for a minimum of 7 days before the experiments. The control rats stayed in their home cage. Partial hepatectomy was performed under general anesthesia (3% isoflurane in O_2_ at 0.6 L/min) in the surgery group. Briefly, the liver was exposed through a 1–2 cm midline abdominal incision. The left lateral lobes of the liver (approximately corresponding to 30% of the organ) were excised. The wound was then infiltrated with 0.25% bupivacaine, and closed by sterile suture. To limit variability, all surgeries were performed by the same person. Animals in RU486 and RU486+CUS+surgery groups were intraperitoneally injected with a daily dose of RU486 (30 mg/kg, dissolved in DMSO) 1 h prior to stress exposure. All procedures were conducted in accordance with the Declaration of the National Institutes of Health Guide for Care and Use of Laboratory Animals and approved by China Medical University Animal Care and Use Committee (No: IACUC-2017001).

### Experimental procedure

Animals received 14-day CUS or CUS with RU486 injection. Twenty-four hours after the last stress session, the rats were subjected to partial hepatectomy under general anesthesia. The body weight was measured every two days during the 14-day CUS. The behavioral changes were evaluated with relatively low-stress methods-open field test and elevated plus-maze test on postoperative days 1, 3 and 7. The rats were euthanized and the hippocampi were harvested for biochemical analyses after the behavioral test. The remaining rats were transcardially perfused with ice-cold saline (0.9%) for 3 min, followed by 4% paraformaldehyde for immunohistochemistry at each time point. The cardiac blood was collected on postoperative days 1, 3 and 7 in the morning (09.00 h).

### Chronic unpredictable stress

Chronic variate stress was adapted from other models of variate stress with modifications [12]. Individual stressors and length of time applied each day are listed in Table 1. In all stress experiments, the stressed rats were exposed to the same order of stressful stimuli. Stress was applied at a different time of each day to minimize predictability. Restraint was carried out by placing the animal in a 21×6 cm plastic tube and adjusting it with a plaster tape on the outside, so that the animal could not move. Forced swimming was carried out by placing the animal in a glass tank with 25 cm of water depth at 23 ± 2°C. The control rats were manipulated every day for 10 min in the home cage to control for nonspecific handing effects.

**Table 1:** Schedule of stressors used during the chronic variate stress treatment.

Day (Time)	Stressor	Time
1 (3:00 P.M.)	restraint	60 min
2 (9:00 A.M.)	forced swimming	15 min
3 (3:00 P.M.)	cold isolation	90 min
4 (5:00 P.M.)	lights on	overnight
5 (8:00 A.M.)	forced swimming	5 min
6 (4:00 P.M.)	water and food deprivation	overnight
7 (2:00 P.M.)	restraint	120 min
8 (3:00 P.M.)	lights off	120 min
9 (9:00 A.M.)	forced swimming	5 min
10(7:00 P.M.)	lights on	overnight
11(2:00 P.M.)	cold isolation	90 min
12(9:00 A.M.)	restraint	60 min
13(6:00 P.M.)	water and food deprivation	overnight
14(8:00 A.M.)	restraint	60 min

### Open-field test

The open-field observation cage consisted of a square wooden arena (100 × 100 × 50 cm) with its inside walls covered in black. It was divided into 25 equal squares, including peripheral area (16 around squares) and central area (9 middle squares). The rats were placed individually into the center of the area and allowed to explore freely. The test was conducted in a quiet room in the morning (8:00–12:00 A.M.). The animals were monitored for 5 minutes with a video tracking software (Smart, San Diego Instruments, San Diego, CA, USA). Spontaneous locomotor activity was assessed by the total amount of distance traveled in the chamber. The time in the central area was taken as measures of anxiety and exploratory behavior.

### Elevated plus-maze test

The elevated plus-maze is an apparatus widely used to evaluate the level of anxiety in rodents. The level of anxiety is measured by variables such as the percentage of time spent and the number of entries in the open arms. Low percentage of time spent and the number of entries in the open arms indicate anxiety. The elevated plus-maze consisted of two open arms (50 cm×10 cm), two enclosed arms (50 cm×10 cm) and a central platform (10 cm×10 cm) made of black plexiglas. The closed arms were surrounded by a 50 cm wall, the open ones presented 0.5 cm edges in order to maximize open-arm entries. The apparatus was elevated at a height of 50 cm above the floor. Environmental temperature was maintained equal to the temperature measured in the housing room. The rats were individually placed at the centre of the maze, facing an open arm and allowed to freely explore the entire maze for 5 min on postoperative days 1, 3 and 7. After each observation, the elevated plus-maze was cleaned with ethyl alcohol (10%) to removes cent cues left from the preceding subject. The time spent on the open and closed arms and the number of entries made into each arm were recorded using a video camera (Sony, DCR-SX44E).

### Plasma GCs concentration

Concentrations of GCs in plasma were quantified by using an enzyme-linked immunoassay (ELISA). In brief, cardiac blood was collected by cardiac puncture into EDTA coated syringes and centrifuged (3000 rpm for 10 min at 4°C). Plasma was collected and stored frozen (-80°C) until assaying. GCs titers were assessed by using a competitive enzyme immunoassay kit, following the manufacturer’s instructions (R&D Systems, Minneapolis, MN).

### Western blot analysis

Hippocampus tissues were collected and homogenized in ice-cold lysis buffer for 30 minutes. The homogenate was centrifuged (12000 g for 10 min 4°C) and the quantity of protein in the supernatants was determined using a BCA protein assay kit (Fdbio science, China). Equal amounts of protein were loaded per well on a 12% SDS-polyacrylamide gel electrophoresis and transferred to PVDF membranes, which were then blocked with 5% skim milk at room temperature for 2 h. Membranes were then incubated using the following antibodies: rabbit monoclonal anti-IL-1β (1:2000; Abcam, Cambridge, UK), mouse polyclonal anti-IL-6 (1:2500; Abcam, Cambridge, UK), rabbit polyclonal anti-BDNF (1:2000; Abcam, Cambridge, UK) overnight at 4°C. The membranes were then incubated in appropriate secondary antibodies diluted in TBST for 2 h at room temperature. Chemiluminescence detection was performed using an ECL Western Blotting kit (cat. # 170–5060; Bio-Rad Laboratones. Inc.). Relative expression levels of protein were normalized by the ratio of target protein (IL-1β, IL-6 and BDNF) to β-actin.

### Real-time PCR

Total RNA from the different treatment conditions was extracted from the whole hippocampus with TRIzol reagent (Takara, Otsu, Japan) according to the manufacturer’s instructions. cDNA was synthesized from total RNA (1.0 μg) using the PrimeScript™ RT reagent Kit with gDNA Eraser (Takara, Otsu, Japan). Two microliters of cDNA were used to perform quantitative real-time PCR. The following primers were used to amplify the mRNA: CD200: 5′-CTGCACACAACTGCATCCTT-3′ (forward) and 5′-GGGCTTTGCTGTAAGTGACC-3′ (reverse); Arg1: 5'-GCAGAGACCCAGAAGAATGG-3' (forward) and 5'-CACGATGTCCTTGGCAGATA-3' (reverse); Glyceraldehyde 3-phosphate dehydrogenase (GAPDH): 5'-GGGGCTCTCTGCTCCTCCCTG-3' (forward) and 5'-AGGCGTCCGATACGGCCAAA-3' (reverse). GAPDH was adopted as an internal control, which were obtained from Sangon Biotech. The reverse transcription reaction was carried out under the following conditions: 95 ˚C for 10 min (initial denaturation), followed by 40 cycles of 95 ˚C for 20 sec, 62 ˚C for 30 sec and 72 ˚C for 30 sec (amplification). The relative gene expression was determined by calculating the expression ratio of the gene of interest to GAPDH. The relative expression of mRNA was quantified using the 2^–ΔΔCt^ method.

### Immunohistochemistry

The sections (5 μm) were pretreated with 3% H_2_O_2_ for 20 min and then incubated with the specific primary antibody overnight at 4°C as follows: goat anti-ionized calcium-binding adaptor protein-1 (Iba1) (1:300; Abcam, Cambridge, UK). An appropriate antibody was applied for 60 min at room temperature. After thoroughly washed, the reaction products were visualized using the DAB method. The sections were counterstained with hematoxylin, dehydrated, and mounted. Control samples were run in parallel omitting the primary antibody. The integrated optical density (IOD) of positively stained area was analyzed at 200× magnificationin CA1 region with image analysis software (Image-Pro Plus 6.0).

### Statistical analysis

All data are presented as the mean ± SEM. Statistical comparisons were subjected to a multivariate analysis of variance (ANOVA) in which stress, surgery and intervention were dependent variables. Bonferroni’s test was employed when ANOVA showed significance. A p-value < 0.05 was considered to be statistically significant.

## Results

### Chronic unpredictable stress decreased the bodyweight of stressed rats

**Fig 1:**
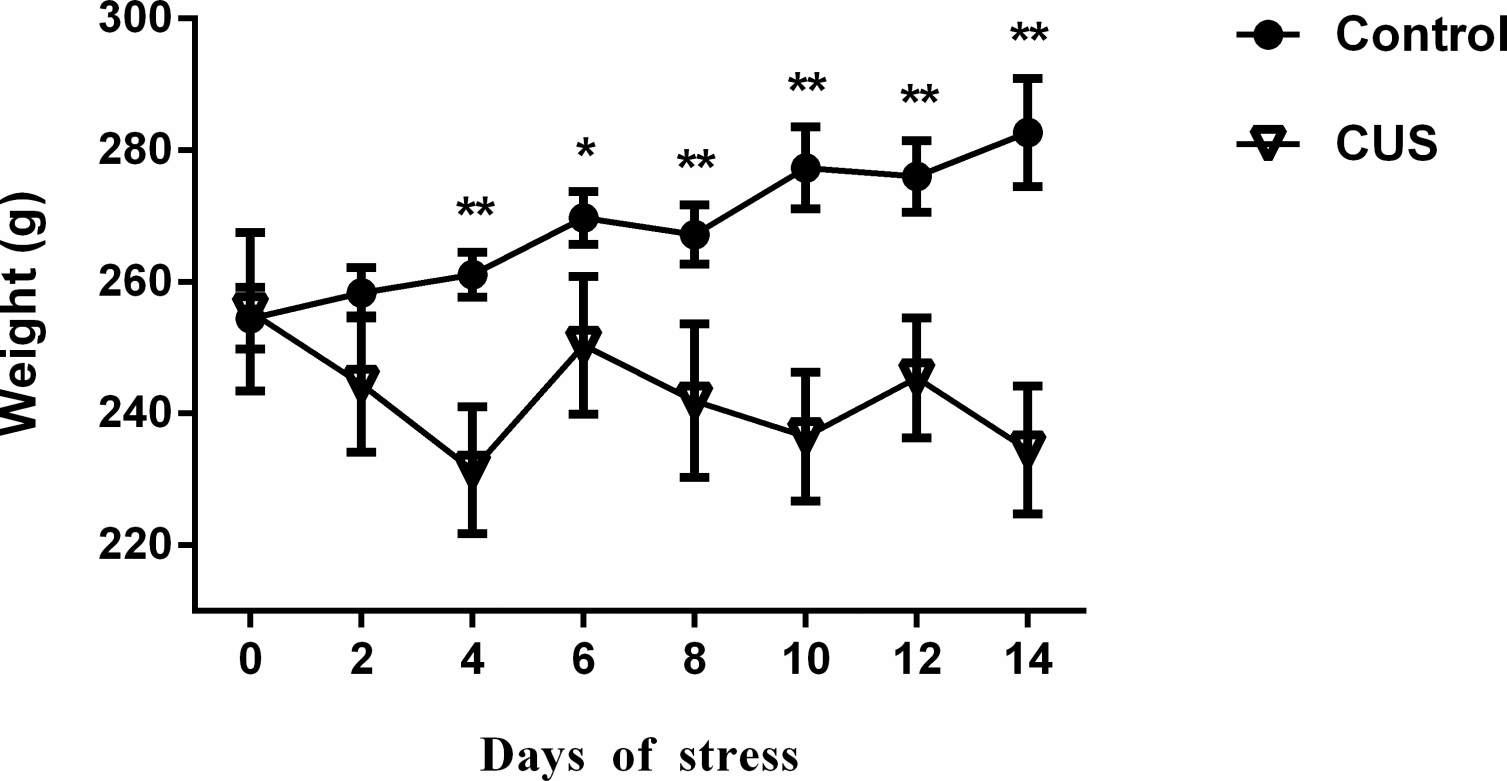
The stressed rats showed decline in body weight over 14 consecutive days of chronic unpredictable stress compared with the controls. The results are represented as the mean ± SEM. *p < 0.05, **p < 0.001 versus the day-matched control group.

CUS exerted a negative effect on the bodyweight gain (Fig 1). While the controls maintained their weight through the protocol, the stressed animals lost 8.15% of weight (p < 0.001) during the 14-day CUS.

### Chronic unpredictable stress exacerbated surgery-induced spontaneous locomotor activity impairment and increased the levels of anxiety

**Fig 2:**
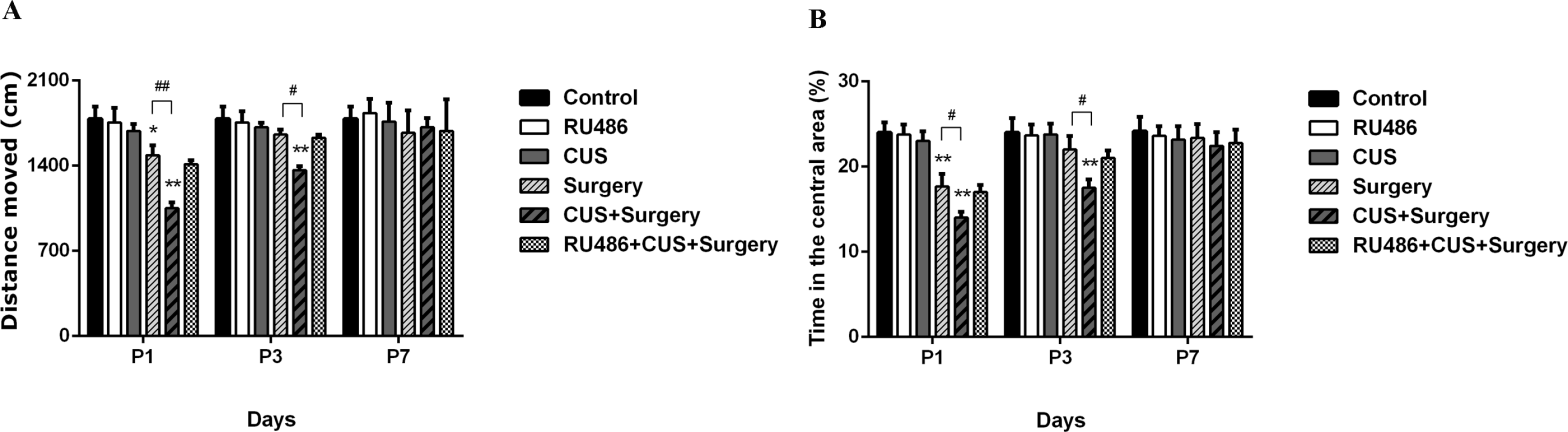
Chronic unpredictable stress exacerbated surgery-induced sickness behavior in the open field test. (A) Total distance moved in the chamber. (B) The time in the central area. The results are represented as the mean ± SEM. *p < 0.05, **p < 0.001 versus the control group; #p < 0.05, ##p < 0.001 versus the surgery group. P1, P3, and P7: postoperative days 1, 3, and 7, respectively.

Two-way ANOVA of the total distance and the time in the central area in the open field test revealed significant effects of CUS (p = 0.010 and p = 0.003, respectively), surgery (p<0.001 and p<0.001, respectively) and CUS×surgery interaction (p = 0.022 and p = 0.017, respectively). There was no significant effect of the CUS on the total distance in the stressed animals compared with the controls 48 h post-stress (p = 0.794). The total distance was shorter in the surgery group compared to that in the control group on postoperative day 1 (p = 0.028). CUS produced an additive effect on the total distance in the surgical animals on postoperative days 1 and 3 (p < 0.001 and p = 0.019, respectively) (Fig 2A). Similarly, the time in the central area in the surgical rats was significantly shorter compared with the controls on postoperative day 1 (p < 0.001). Significant difference of the time in the central area was also observed between the surgery group and the CUS+surgery group on the postoperative days 1 and 3 (p = 0.021 and p = 0.003, respectively) (Fig 2B). When pretreated with RU486, the distance moved was similar between the surgery group and the RU486+CUS+surgery group. A similar pattern was observed for the time in the central area. RU486 alone did not depress locomotor activity and alter anxiety levels.

**Fig 3:**
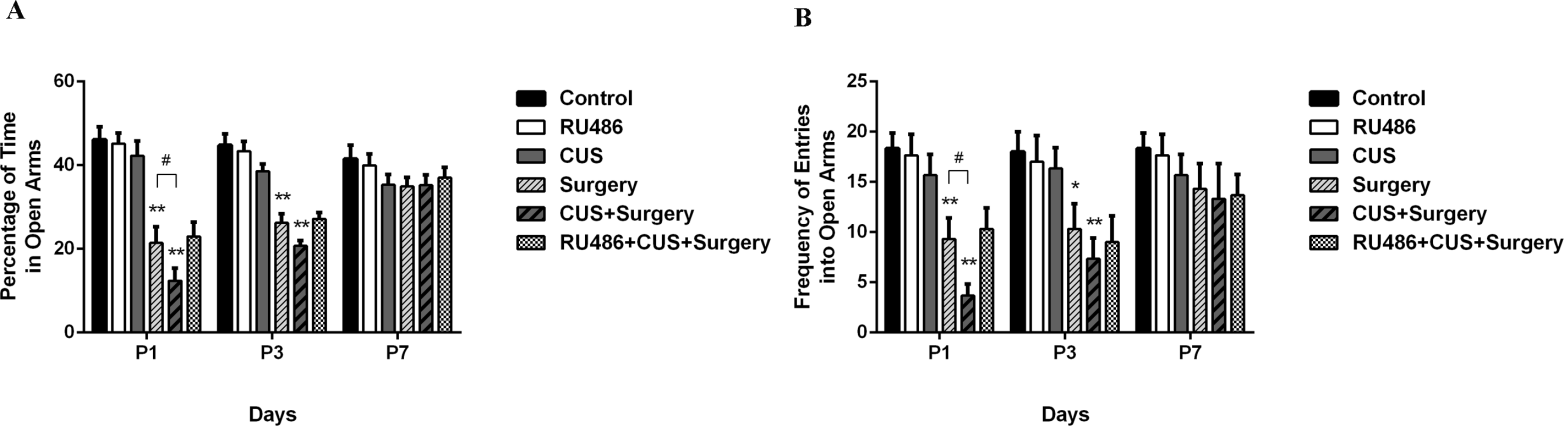
Surgical trauma significantly decreased open arms exploration in the elevated plus-maze test on postoperative days 1 and 3. Chronic unpredictable stress enhanced surgery-induced sickness behavior on postoperative day 1. (A) Percentage of time in open arms. (B) Frequency of entries into open arms. The results are represented as the mean ± SEM. *p < 0.05, **p < 0.001 versus the control group; #p < 0.05 versus the surgery group. P1, P3, and P7: postoperative days 1, 3, and 7, respectively.

Analysis of the percentage of time spent and the number of entries in the open arms in the elevated plus-maze test revealed significant effects of CUS (p = 0.021 and p<0.001, respectively), surgery (p<0.001 and p<0.001, respectively), and CUS+surgery interaction (p = 0.031 and p = 0.011, respectively). Compare to the controls, surgical trauma significantly decreased the percentage of time spent (p < 0.001 and p < 0.001, respectively) and the number of entries (p < 0.001 and p = 0.002, respectively) in the open arms on postoperative days 1 and 3 (Fig 3A and 3B). CUS or RU486 alone failed to significantly affect the percentage of time spent and the number of entries in the open arms. However, CUS produced an additional decrease in the open arm exploration in the surgical rats on postoperative day 1 (p = 0.003 and p = 0.036, respectively). When pretreated with RU486, the percentage of time spent (p = 0.992 and p = 0.989, respectively) and the number of entries (p = 0.726 and p = 0.998, respectively) in the open arms was similar between the surgery group and the RU486+CUS+surgery group on postoperative days 1 and 3 (Fig 3A and 3B).

### Chronic unpredictable stress exaggerated surgery-induced neuroinflammatory responses in the hippocampus

**Fig 4:**
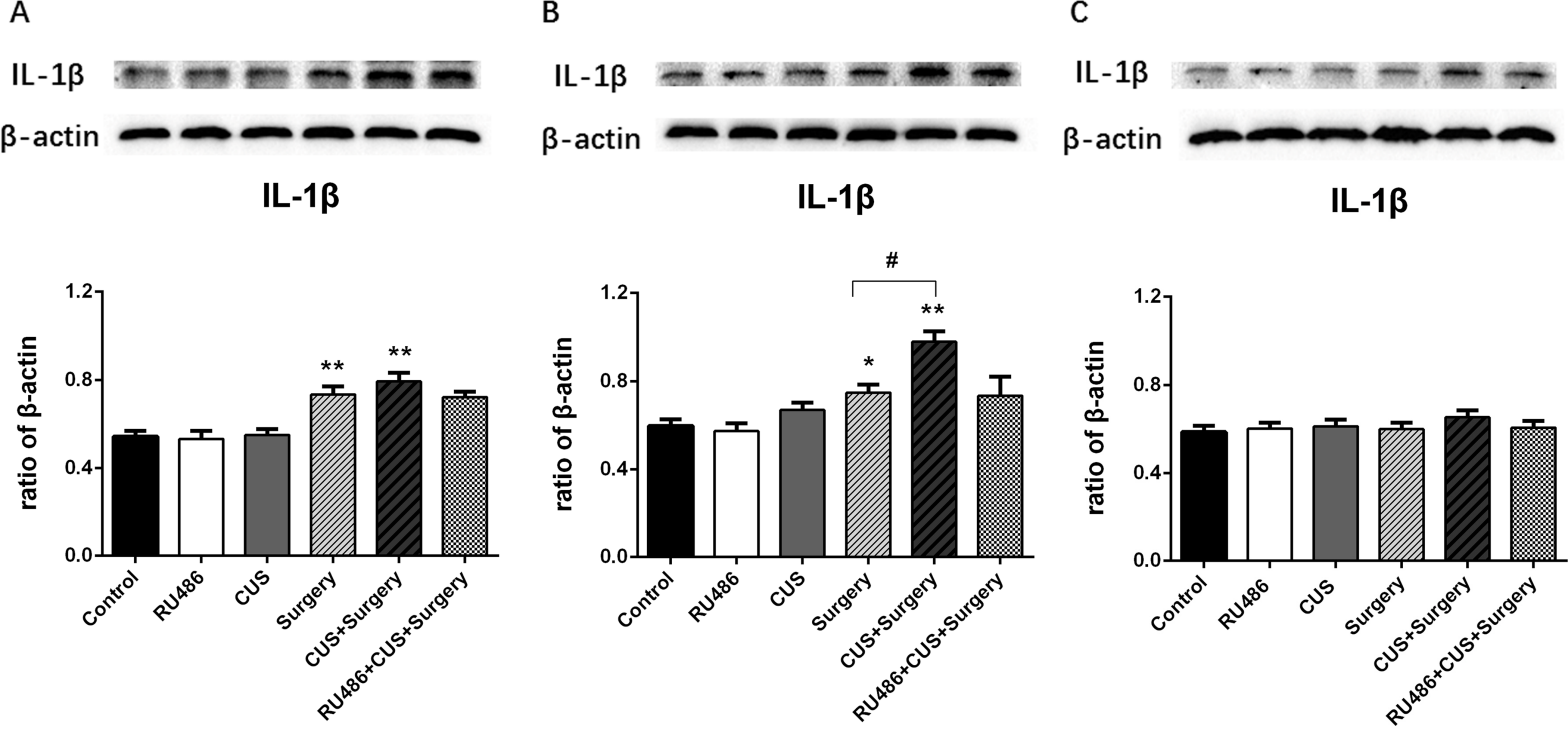
Compared with the surgery group, chronic unpredictable stress (CUS) amplified surgery-induced hippocampal IL-1β expression on postoperative day 3. Pretreatment with RU486 significantly blunted the potentiating effects of CUS on surgery-induced IL-1β levels in the brains of adult rats. (A) Postoperative day 1. (B) Postoperative day 3. (C) Postoperative day 7. The results are represented as the mean ± SEM. *p < 0.05, **p < 0.001 versus the control group; #p < 0.05 versus the surgery group.

**Fig 5:**
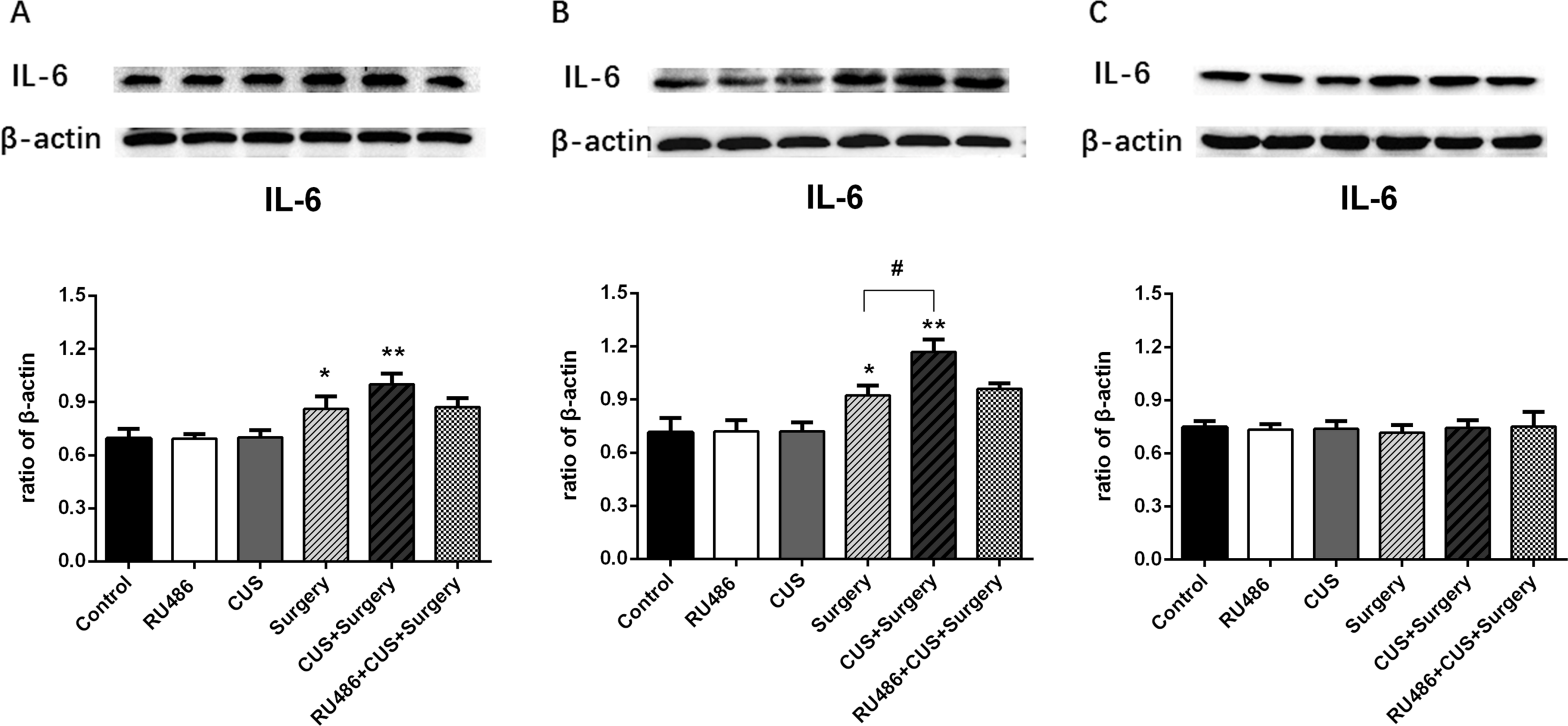
Chronic unpredictable stress (CUS) aggravated surgery-induced hippocampal IL-6 expression on postoperative day 3. Pretreatment with RU486 significantly blunted the potentiating effects of CUS on surgery-induced IL-6 levels in the brains of adult rats. (A) Postoperative day 1. (B) Postoperative day 3. (C) Postoperative day 7. The results are represented as the mean ± SEM. *p < 0.05, **p < 0.001 versus the control group; #p < 0.05 versus the surgery group.

Surgical trauma significantly increased the levels of IL-1β and IL-6 on postoperative days 1 (p < 0.001 and p = 0.033, respectively) and 3 (p = 0.03 and p = 0.011, respectively). In the absence of surgical challenge, CUS failed to alter the levels of pro-inflammatory cytokines. However, CUS amplified surgery-induced pro-inflammatory cytokine IL-1β expression on postoperative day 3 (p = 0.002), and IL-1β expression returned to baseline on day 7 (p = 0.248) compared with the day-matched surgery group (Fig 4). RU486 alone failed to significantly alter hippocampal pro-inflammatory cytokine levels when compared to the naive controls at any time point. However, administered with RU486 (30 mg/kg) prior to CUS and surgery, the levels of IL-1β were similar in the RU486+CUS+surgery group and the surgery group. A similar pattern was observed for IL-6 protein (Fig 5). This indicated that RU486 substantially blunted the potentiating effects of CUS on surgery-induced pro-inflammatory processes in the hippocampus.

### Chronic unpredictable stress upregulated surgery-induced microglial Iba-1 expression and reduced the mRNA levels of M2 phenotype marker Arg1

**Fig 6:**
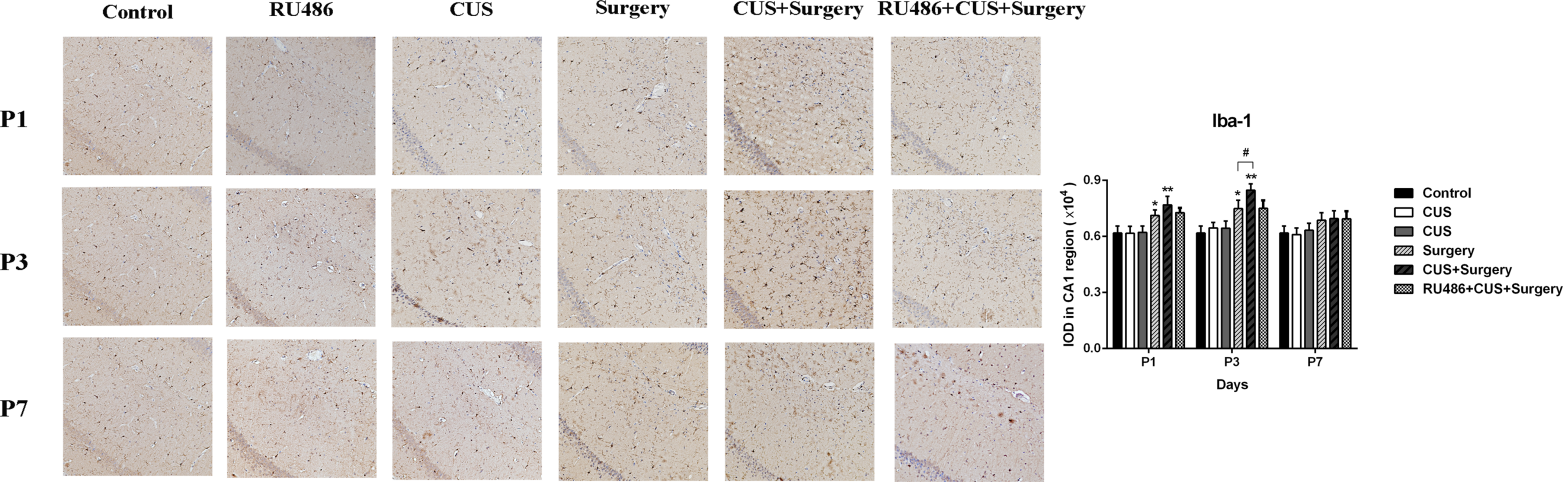
Chronic unpredictable stress (CUS) upregulated surgery-induced microglial Iba-1 expression on postoperative day 3 and the Iba-1 expression improved on day 7 compared with that of the surgery group. Pretreatment with RU486 significantly blunted the potentiating effects of CUS on surgery-induced Iba-1 levels on postoperative day 3 in the adult rats. The results are represented as the mean ± SEM. *p < 0.05, **p < 0.001 versus the control group, #p < 0.05 versus the surgery group. P1, P3, and P7: postoperative days 1, 3, and 7, respectively.

Compared to the naive controls, CUS failed to increase the expression of Iba-1 48 h post-stress. The levels of Iba-1 were significantly upregulated compared with the controls on postoperative days 1 (p = 0.037) and 3 (p = 0.002), and returned to baseline on day 7 (p = 0.209). Higher levels of Iba-1 were observed in the animals of CUS+surgery group compared with those of the surgery group on postoperative day 3 (p = 0.027). Pretreatment with RU486 blocked the effects of CUS on surgery-induced Iba-1 upregulation (Fig 6).

**Fig 7:**
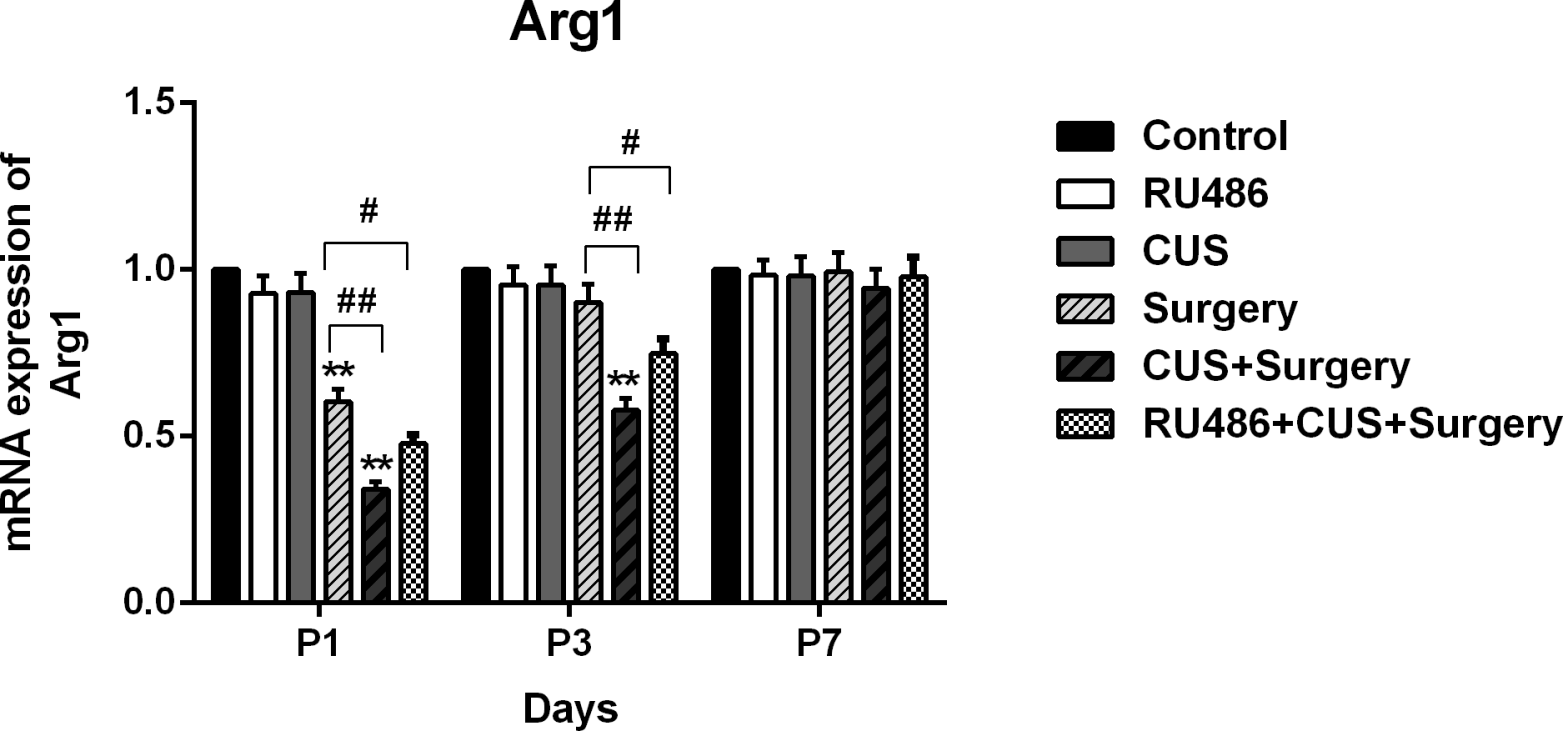
Compared with the control group, the mRNA levels of M2 phenotype marker Arg1 were significantly downregulated on postoperative day 1 in both surgery group and CUS+surgery group and recovered to basal level on day 7. An additional significant decrease in hippocampal Arg1 expression was observed in CUS+surgery group compared to the surgery group on postoperative days 1 and 3. Treatment with RU486 partially blunted the effects of CUS on surgery-induced downregulation of Arg1 expression. The results are expressed as the mean ± SEM. **p < 0.001 versus the control group; #p < 0.05, ##p < 0.001 versus the surgery group. P1, P3, and P7: postoperative days 1, 3, and 7, respectively.

CUS failed to downregulate the levels of microglial M2 phenotype marker Arg1 in the stressed rats compared with the controls. The gene levels of Arg1 were significantly decreased following surgical challenge on postoperative day 1 (p < 0.001). CUS produced an additional reduction of Arg1 expression in the CUS+surgery group compared with the surgery group on postoperative days 1 and 3 (p < 0.001 and p < 0.001, respectively). Treatment with RU486 partially blunted the potentiating effects of CUS on surgery-induced downregulation of Arg1 expression in the hippocampus (Fig 7).

### Chronic unpredictable stress and surgical trauma decreased the levels of CD200 mRNA in the hippocampus

**Fig 8:**
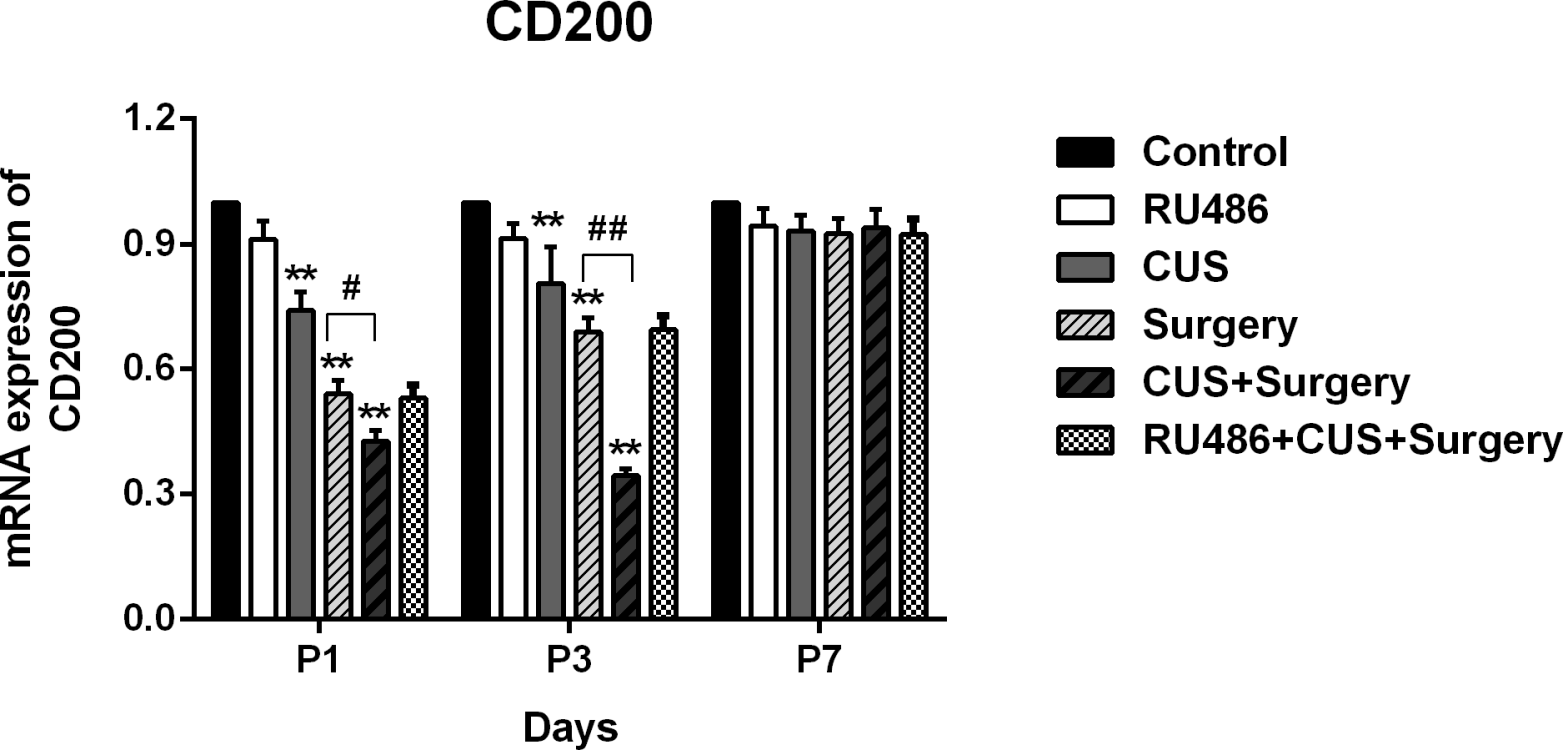
Hippocampal CD200 mRNA was downregulated on postoperative days 1, 3 and improved on day 7 in the surgical rats. CUS had an additional significant decrease in hippocampal CD200 mRNA expression on postoperative days 1 and 3. Administration of RU486 attenuated the effects of stress on surgery-induced downregulation of CD200 expression. The results are represented as the mean ± SEM. **p < 0.001 versus the control group; #p < 0.05, ##p < 0.001 versus the surgery group. P1, P3, and P7: postoperative days 1, 3, and 7, respectively.

Compared to the controls, CD200 mRNA expression was downregulated in the CUS group at 48 and 96 h post-stress (p < 0.001 and p < 0.001, respectively). The levels of CD200 mRNA was significantly decreased on postoperative days 1 and 3 (p < 0.001 and p < 0.001, respectively), and improved on day 7 (p = 0.128). An additional significant decrease in hippocampal CD200 mRNA expression was observed when partial hepatectomy was performed in the stressed animals on postoperative days 1 (p = 0.007) and 3 (p < 0.001). Administration of RU486 attenuated the potentiating effects of CUS on surgery-induced downregulation of CD200 expression (Fig 8).

### The combination of chronic unpredictable stress and surgical trauma reduced the levels of BDNF in the brain

**Fig 9:**
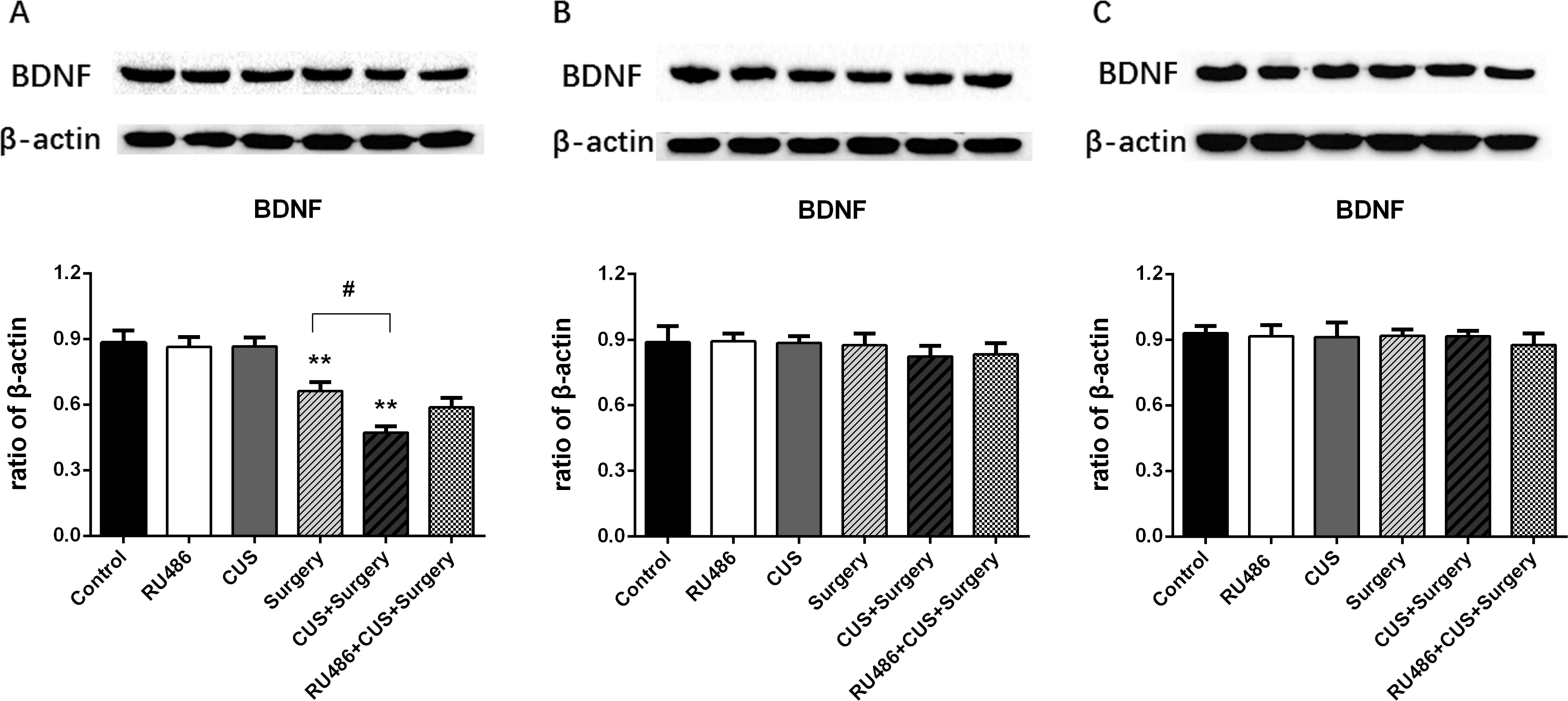
The combination of chronic unpredictable stress (CUS) and surgical trauma reduced the levels of BDNF in the brain on postoperative day 1. Pretreatment with RU486 significantly blunted the potentiating effects of CUS on surgery-induced BDNF levels in the brains of adult rats. (A) Postoperative day 1. (B) Postoperative day 3. (C) Postoperative day 7. The results are represented as the mean ± SEM. **p < 0.001 versus the control group; #p < 0.05 versus the surgery group.

Surgical trauma significantly decreased the levels of BDNF compared with the controls on postoperative day 1 (p < 0.001), while CUS alone failed to downregulate BDNF expression in the stressed rats 48 h post-stress (Fig 9A). Furthermore, the lower levels of BDNF were observed in the CUS+surgery group compared with the surgery group on postoperative day 1 (p = 0.002) (Fig 9A). Pretreatment of RU486 blunted these priming effects induced by CUS (Fig 9A).

### Chronic unpredictable stress and surgical trauma increased the levels of plasma GCs

**Fig 10:**
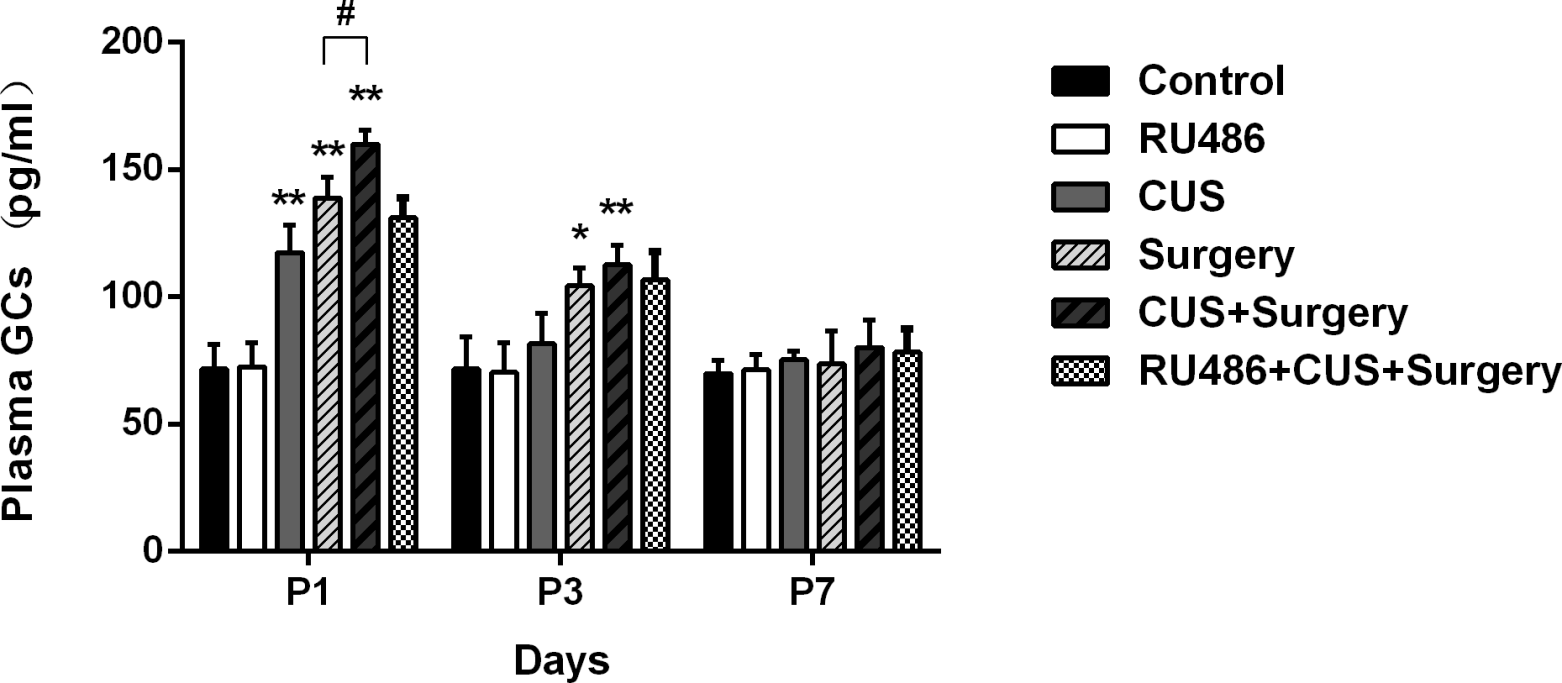
Surgical trauma significantly increased the levels of glucocorticoids (GCs) on postoperative days 1 and 3 compared with the controls. Chronic unpredictable stress enhanced surgery-induced the increased levels of GCs on postoperative day 1. The results are represented as the mean ± SEM. *p < 0.05, **p < 0.001 versus the control group, #p < 0.05 versus the surgery group. P1, P3, and P7: postoperative days 1, 3, and 7, respectively.

ELISA analysis showed that exposure to CUS resulted in a significant increase in plasma GCs compared to the non-stressed rats 48 h (p < 0.001) after the last session of stress and the levels of GCs returned to baseline 96 h post-stress (Fig 10). Surgical trauma significantly increased the levels of plasma GCs on postoperative days 1 and 3 (p < 0.001 and p = 0.002, respectively) and returned to baseline on day 7 compared with the controls. CUS enhanced the increased levels of GCs on postoperative day 1 (p < 0.001) (Fig 10).

## Discussion

Accumulating evidence has shown that both acute and chronic stress potentiated the sickness response to LPS administered 24 h post-stress [13–15]. In the present study, surgical trauma impaired spontaneous locomotor activities and increased the levels of anxiety in the adult rats. CUS exacerbated surgery-induced sickness behavior. There effects were blocked by pretreatment with RU486, indicating that GCs, at least partly, is involved in the stress-induced sensitization of behavior changes following surgical challenge. Importantly, these changes were accompanied by microglial activation and neuroinflammatory responses in the brain, which might mediate sickness behavior.

The severity of the surgery influences the magnitude of the immune response and has been shown to correlate with the degree of postoperative inflammation and sickness behavior [16, 17]. Previous work has shown that the incidence of POCD in elderly patients after minor surgery (primarily laparoscopy) was significantly lower than those after cardiac and noncardiac major surgery suggesting that extent of surgery contributes to postoperative brain dysfunction [18]. A study by Hempenius et al also indicates that the severity of the surgical procedure is independent risk factors for postoperative delirium in elderly patients undergoing elective surgery [19]. Additionally, Rosczyk et al have demonstrated that locomotor activity is not depressed in both adult and aged mice following sham operation-minor abdominal surgery, which reveals that the decrease in locomotion is not due to minor surgery procedure [20]. It is likely that a more “major” surgery-partial hepatectomy would induce a state of neuroinflammation that could result in sickness behavior.

Pro-inflammatory cytokines inhibit hippocampal neuronal functions, including long-term potentiation (LTP) and dendritic branching, which are involved in memory formation and maintenance [21]. Stressors can induce two different types of inflammatory responses in the brain. The first is a rapid, short duration (several hours) increase in inflammatory mediators [22]. Brain levels of pro-inflammatory cytokines are elevated immediately after a moderate duration (2 h) of stress and persist for 4–6 h [23]. The second is a slower developing, longer lasting (days) sensitization (or ‘‘priming”) of neuroinflammatory responses to subsequently occurring infectious/pathogenic stimuli or stressors (i.e. delayed challenge) [13]. Acute and chronic stress has been found to sensitize neuroinflammatory responses to both peripheral and central immunologic challenges [13, 24, 25]. CUS alone failed to modulate the expression of pro-inflammatory cytokines 48 h post-stress. However, consistent with a growing body of evidence, this study demonstrated the priming effects of CUS in neuroinflammatory processes [26]. CUS amplified surgery-induced neuroinflammatory responses in the brain. Additionally, Barrientos et al demonstrated a single intracisternal administration of IL-1 receptor antagonist (IL-1RA) at the time of surgery was sufficient to block both the behavioral deficit and the neuroinflammatory response. Injecting the same dose of IL-1RA peripherally failed to have a protective effect [27]. These data provided strong support for the specific role of central, not peripheral, IL-1β in behavior deficits.

Microglia are primary immune cells and major source of pro-inflammatory cytokines in the central nervous system (CNS) [28, 29]. Espinosa-Oliva et al found that microglia were neuroimmune substrates of peripheral stress-induced GCs action, which in turn ‘‘prime” microglia to over-respond to subsequent challenge in the aged brain [24]. Notably, hippocampal microglia isolated 24 h post-stress also exhibited a potentiated neuroinflammatory response to LPS ex vivo, suggesting that central microglia instead of peripheral immunologic substrate(s) were sensitized or primed. In this study, CUS sensitized microglia to pro-inflammatory immunophenotype. When further stimulated in this state through a peripheral surgical challenge, microglia overexpressed pro-inflammatory cytokines (e.g., IL-1β and IL-6). These data suggest that stress-induced microglial priming contributes to neuroinflammatory responses to subsequent surgical challenge.

On the basis of gene expression profiles, activated microglia may be categorized into two opposite types: M1 phenotype and M2 phenotype [30]. M1 represents a detrimental state of microglia, characterized by high expression of pro-inflammatory cytokines (e.g. IL-1β, IL-6 and TNF-α). Conversely, M2 phenotype may reverse the neuron loss, repair neural networks and enhance the production of anti-inflammatory cytokines (e.g. IL-10) and neurotrophic mediators (e.g. BDNF) [30]. M1 and M2 polarization states of microglia play an important role in controlling the balance between pro-inflammatory and anti-inflammatory conditions. This study demonstrated that CUS exaggerated surgery-induced neuroinflammatory responses and exacerbated sickness behavior by inhibiting the function of M2-polarized microglia. CUS mediated the shift of the neuroimmune microenvironment toward a microglial M1 phenotype.

CD200 expressed on the surface of neurons is thought to maintain microglial cells in a quiescent state through interactions with its receptor CD200R [31]. The CNS parenchyma shows high levels of CD200 as well as very low levels of basal MHC-II and Iba-1, indicating the quiescent immunophenotype of the normal brain microenvironment [32]. The present study demonstrated that CUS downregulated hippocampal CD200 expression at 48 and 96 h post-stress. The effect of CUS on CD200 suggests that stress-induced sensitization of microglia may be mediated in part by attenuation of neuronal control of microglia.

GCs appear to play a pivotal role in CUS-induced potentiation of neuroinflammatory processes [9, 33]. GCs modulate innate immune signaling pathways (e.g., Toll-like receptors and inflammasome formation) that are pivotal to generating a pro-inflammatory immune response [34]. In this study, treatment with GCs receptor antagonist RU486 blocked CUS-induced priming of hippocampus pro-inflammatory responses and microglial activation following surgical procedure. These data suggest that elevated GCs may mediate CUS-induced sensitization of neuroinflammatory processes triggered by surgical trauma.

Human studies provide direct evidence of cognitive impairment following high levels of GCs. A 4-day treatment with dexamethasone produced a selective impairment of declarative memory [35]. Cushing’s syndrome is associated with cognitive impairment and hippocampal atrophy that is reversible following the lowering of cortisol levels after treatment [36]. Consistent with previous research, this study demonstrated that surgical trauma significantly increased the levels of serum GCs, which related to sickness behavior [37]. Thus, a body of knowledge suggests that GCs, at least partly, involves behavior deficits in a variety of settings.

Increased levels of GCs are almost universally considered to be anti-inflammatory [38]. However, the results in this study appeared contradictory. Stress-induced GCs failed to suppress neuroinflammatory responses following surgical procedure. Of particular relevance to the present study, the timing of stress exposure relative to an immune challenge was a critical parameter in determining the outcome. Frank et al found that GCs treatment prior to LPS potentiated the neuroinflammatory response, whereas GCs treatment after LPS blunted neuroinflammatory responses to LPS. Notably, Barnum et al showed that CUS as well as a chronic psychological stress blunted the neuroinflammatory response to LPS [39]. It is important to note that LPS was administered two weeks after the last stress. Whereas consistent with other studies, partial hepatectomy was performed 24 h post-stress in this study [7]. The timing of immunologic challenge relative to stressor offset may account for these discrepant findings.

GCs bind two different receptors: high-affinity mineralocorticoid receptor (MR) and the lower-affinity GR in the CNS. MR is heavily occupied basally and becomes saturated by GCs levels in the mild stress range, whereas GR is heavily occupied only after major stressors [8]. Because MR and GR signaling can have different transcriptional effects, basal and high-stress GCs levels can have divergent, even opposite effects [40]. Pretreatment with RU486 blocked CUS-induced microglia activation and neuroinflammatory responses in the brain, which indicated that GCs and GR involved in the deleterious effects of CUS.

BDNF is an important regulator of synaptic transmission and LTP in the brain, which is associated with learning and memory formation [41, 42]. Administered neural injections of a BDNF antibody exacerbated cognitive deficits assessed by a Morris water maze [43]. Contrarily, exogenous BDNF improved the cognitive performance [44]. A growing body of evidence suggests that severe stress can suppress BDNF signaling, impair synaptic activity and increase susceptibility to affective disorders, resulting in neuronal atrophy and cognitive impairment [45]. Several evidence indicate that chronic stress and low level of BDNF are the major components of sickness behavior [46]. In this study, surgical trauma in combination with CUS inhibited BDNF expression in the brain, which was accompanied with sickness behavior. Surgery-induced behavioral deficits were mediated, in part, by downregulation of hippocampal BDNF expression.

Stress affects neuroimmune system functions both directly and indirectly. Previous research has indicated that chronic stress induces inflammatory responses, cognitive impairments and regulates microglial activity in the brain region [47, 48]. However, in this study, CUS alone failed to exacerbate sickness behavior, modulate the expression of pro-inflammatory cytokines and alter microglial Iba-1 and Arg 1 expression 48 h post-stress. Contradictory results may be considered to be due to different treatment protocols, such as animals (adult vs aged rats), stress variables [e.g., duration (14 vs 40 days) and frequency] and time point of evaluation (48 vs 2 hours) [7]. Furthermore, Bian et al provides evidence that CUS-induced impairments in spatial learning and memory and CUS-induced changes in glia cells could be reversible [49].

This study provides converging evidence that CUS reinforces the effect of surgical trauma challenges in a synergistic manner, creating an exaggerated sickness behavior and neuroinflammatory responses and inhibiting BDNF expression in this rodent model. Stress-induced microglial activation contributes to the sensitization of pro-inflammatory responses. GCs play a pivotal role in enhancing stress-induced neuroinflammatory responses by modulation of microglia functions.

## Supporting information

S1 Checklist NC3Rs ARRIVE guidelines checklist. (PDF)

S1 Dataset Relevant data underlying the findings described in manuscript. (XLS)
